# Moderate alcohol consumption does not protect cognitive function when controlling for income and cultural factors

**DOI:** 10.3389/fnagi.2025.1569069

**Published:** 2025-05-26

**Authors:** Kerri M. Gillespie, Eva Kemps, Melanie J. White, Selena E. Bartlett

**Affiliations:** ^1^Faculty of Health, School of Clinical Sciences, Queensland University of Technology, Kelvin Grove, QLD, Australia; ^2^College of Education, Psychology and Social Work, Flinders University, Adelaide, SA, Australia; ^3^Faculty of Health, School of Psychology and Counselling, Queensland University of Technology, Kelvin Grove, QLD, Australia

**Keywords:** cognition, alcohol, socioeconomic status, income, language

## Abstract

**Introduction:**

Alcohol consumption is commonly considered to be a modifiable risk factor in cognitive decline. However, numerous studies have found an association between light to moderate consumption of alcohol and enhanced cognitive function. It has been proposed that this finding is due to the effects of socioeconomic status (SES) or other covariates on drinking behaviors. The present study aimed to investigate the effect of alcohol on cognition, and the impact of different socioeconomic indicators on this relationship.

**Methods:**

An online, cross-sectional survey, including an assessment of five domains of cognitive function, was conducted in 123 healthy adults between 18 and 70 years of age. Secondary analysis of the 2018 National Health Survey was conducted to investigate drinking patterns, and their relationships to SES indicators, in the Australian population.

**Results:**

Income and education showed dissimilar patterns of association with alcohol consumption. Enhancements in cognitive function were associated with light to moderate dose and frequency of alcohol consumption when unadjusted, or adjusted for SES using education level as an indicator. Benefits of light to moderate dose and frequency of consumption were not evident when using income as an indicator for SES. Inclusion of language spoken in the home as a covariate also resolved any association between moderate consumption and enhanced cognitive function.

**Discussion:**

Findings suggest that associations between moderate alcohol consumption and cognition are an artifact of income, language proficiency, and culture. The use of income is more indicative of drinking behavior than education, and should be controlled for in studies of alcohol consumption behavior. Language spoken at home is also an important consideration as this factor is significantly associated with income, alcohol consumption, and cognitive test performance.

## 1 Introduction

Alcohol is considered a modifiable risk factor for cognitive decline. Excessive consumption can lead to changes in the brain similar to those seen in aging, with significant reductions in gray and white matter volume (Squeglia et al., 2014; Guerri and Pascual, 2019; Daviet et al., 2022). Large cohort, biobank, and brain imaging studies have found even light consumption associated with reductions in cortical volume and increased risk of dementia (Topiwala et al., 2017; Immonen et al., 2020; Daviet et al., 2022; Zheng et al., 2024). However, a large number of epidemiologic studies have identified an association between moderate, regular alcohol consumption and a decreased risk for cognitive decline and dementia (den Heijer et al., 2004; Almeida et al., 2014; Reas et al., 2016; Xu et al., 2017; Zhang et al., 2020; Akagi et al., 2022; Jeon et al., 2023).

Reasons for these conflicting findings have been proposed, but are still unclear. Stockwell et al. (2024) reviewed studies that associated lower mortality risk with low to moderate alcohol consumption and found they were more likely to rate alcohol consumption based on a short retrospective period (days or months); include older participants (aged over 55 years); include ex-drinkers in the non-drinking category; and control for smoking and socioeconomic status (SES). Smoking has been suggested as a mediator for alcohol use, and its complex relationship to alcohol and health may make it inappropriate to include as a covariate (Stockwell et al., 2024). Naimi et al. (2017) highlight the fact that those who stop drinking tend to have worse overall health, further biasing current non-drinkers toward ill health.

Socioeconomic status, and how it is measured, is another mechanism for potential bias (Towers et al., 2016; Gómez et al., 2021). Population survey data suggests that people with lower SES drink infrequently but in higher volumes than their higher SES counterparts [Centers for Disease Control and Prevention [CDC], 2012], while higher SES individuals frequently consume low or moderate amounts of alcohol (Casswell et al., 2003; Platt et al., 2010; Towers et al., 2016), biasing moderate drinkers toward improved health and cognition. A study of younger adults found drinking was positively associated with income, educational attainment, and health (Ng Fat and Shelton, 2012). Wine in particular has been associated with reduced cognitive risk (Neafsey and Collins, 2011; Xu et al., 2017), and is also associated with greater intelligence, more education, and higher SES (Mortensen et al., 2001). One proposed mechanism is via an improvement in cardiovascular health due to moderate alcohol intake (Ronksley et al., 2011). However, suggested cardioprotective effects of alcohol may also be an artifact of SES. Norström and Landberg (2023) identified that increased alcohol consumption led to increased ischemic heart disease mortality, but only in low SES groups.

A common indicator of SES is educational achievement or years of education, which may be a poor gauge of SES (Darin-Mattsson et al., 2017). Education has frequently shown a weaker association with health in later life than income (Darin-Mattsson et al., 2017). Tertiary education is becoming increasingly common, biasing older participants toward a lower education classification (Galobardes et al., 2006). Education and income also show different patterns of association with alcohol consumption. A population-based study in Japan (Murakami and Hashimoto, 2019) found low educational attainment was associated with greater risk of heavy drinking, while lower income was associated with a lower risk.

Cultural and linguistic background may also bias findings. The Australian Bureau of Statistics (ABS) (Australian Bureau of Statistics, 2011) found that citizens born in Australia who spoke a language other than English in the home had higher levels of education than those speaking mostly English. Individuals from a culturally and linguistically diverse (CALD) background are also less likely to drink alcohol (Australian Institute of Health and Welfare, 2024) and have lower incomes (Wang et al., 2023). Studies have also found significant impacts of language proficiency and multilingualism on cognitive test performance (Cormier et al., 2022; Pacifico et al., 2023). Despite the strong relationship between CALD membership, alcohol consumption, SES, and cognitive test outcomes, language spoken in the home and English language proficiency are rarely, if ever, controlled for in studies investigating the impacts of alcohol consumption on cognitive function.

The aim of this study was to examine the relationships between patterns of alcohol consumption and cognitive function, and investigate how these relationships are influenced by covariates. We therefore evaluated the impacts of alcohol dose and consumption frequency on neurocognitive domains. The differential effects of adjusting for covariates relating to education, income, and language was then investigated. Data from the 2018 National Health Survey were used to support these findings by investigating the relationships between these covariates in the Australian population.

## 2 Materials and methods

### 2.1 Study design and sample description

This cross-sectional study was reported in accordance with the Strengthening the Reporting of OBservational studies in Epidemiology (STROBE) reporting guidelines (Cuschieri, 2019). The study incorporated online, quantitative data collection to capture data for our “cognition study.” The recruitment period was between the 12*^th^* of June and 17*^th^* of November, 2023. Individuals were recruited if they were between 18 and 70 years of age, were able to read and write English, and had the use of a computer, laptop, or tablet that could access the internet. Individuals were not able to participate if they had a current or previous major psychiatric illness, substance use disorder, or eating disorder, or were experiencing a serious health condition or taking medications that influenced their thinking, diet, blood sugar levels, or weight. The study recruited 144 healthy adults from the community. Two of these were flagged as suspicious by the survey software (Qualtrics) and a further 14 were removed for failing to complete the cognitive assessment. Five participants not residing in Australia were removed to enable comparable analysis of income and socioeconomic status, leaving 123 participants in the final sample (see Table 1).

**TABLE 1:** Sample descriptives.

Items	All participants
Age *^Mean (SD)^*	37.85 (14.58)
**Gender *^N (%)^***	
Male	33 (26.8)
Female	90 (73.2)
**Primary language spoken at home *^N (%)^***	
English	100 (81.3)
Other	23 (26.8)
**Employment *^N (%)^***	
Full time	47 (38.2)
Part time	26 (21.1)
Student	38 (30.9)
Homemaker/retired	8 (6.5)
Unemployed	4 (3.3)
**Highest educational qualification *^N (%)^***	
Postgraduate degree	56 (45.5)
Undergraduate degree	39 (31.7)
Trade/technical/vocational training	7 (5.7)
Grade 12	21 (17.1)
**Household income *^N (%)^***	
Under $30,000	16 (13.0)
$30,100–$50,000	19 (15.4)
$50,100–$70,000	9 (7.3)
$70,100–$110,000	21 (17.1)
$110,100–$150,000	31 (25.2)
$150,100–$200,000	13 (10.6)
Over $200,000	14 (11.4)
**Ethnicity *^N (%)^***	
Caucasian	83 (67.5)
First Nations People	2 (1.6)
Asian	31 (25.2)
Other	7 (5.7)
**Alcohol consumption frequency *^N (%)^***	
Never or rarely	53 (43.1)
Infrequent (more than once per month, less than once per week)	29 (23.6)
1–2 times per week	28 (22.8)
3–7 days per week	13 (10.6)
**Alcohol dose (standard drinks per week) *^N (%)^***	
Abstinent (less than 12 drinks per year)	41 (33.3)
Light-moderate (up to 7 drinks per week for women, and 14 for men)	69 (56.1)
Heavy (more than 7 per week for women and 14 drinks for men)	13 (10.6)
**Neurocognitive domain score *^Mean (SD)^***	
Global cognition	483.21 (111.68)
Reasoning	576.99 (126.21)
Attention	501.49 (158.22)
Memory	497.82 (154.21)
Perception	471.13 (101.34)

The National Health Survey (NHS) is a country-wide household-based health survey conducted every 3–6 years by the Australian Bureau of Statistics (Australian Bureau of Statistics, 2018). We conducted secondary analysis of weighted data from 16,370 adults included in the 2018 NHS.

### 2.2 Ethical statement

This study was approved by the Queensland University of Technology Human Research Ethics Committee (Ethics ID: 5872). All participants gave written, informed consent, before participating in online surveys.

### 2.3 Sample size calculation

A sample size calculation was conducted using G*Power software for linear multiple regression (fixed model, R2 deviation from zero). To detect a medium effect size (f2 = 0.15) with 80% power, alpha error probability of α = 0.05, and seven predictors, a minimum sample size of 103 was required.

### 2.4 Data collection

Participants in the cognition study were asked to complete all online assessments on the same day, on their home computer or other device. NHS data was collected by trained interviewers who surveyed the residents of 16,384 randomly chosen dwellings. Methods and survey composition is described in detail online (Australian Bureau of Statistics, 2018).

### 2.5 Measures

#### 2.5.1 General cognitive assessment battery (GCAB) by Cognifit™

The Cognifit™ GCAB is a widely used and validated (Peretz et al., 2011; Yaneva et al., 2022), online cognitive assessment program that has demonstrated good internal consistency on each measure of cognitive function (α = 0.571 to α = 1) and test-retest reliability (0.696–0.998) (Cognifit, 2022). The online assessment lasts approximately 30 min. Raw data from 21 cognitive functions are age-adjusted by Cognifit and converted to five domains (reasoning, attention, memory, perception, and coordination) and a global score, scored from 0 to 800.

#### 2.5.2 Alcohol consumption

Participants were asked to rate their frequency of alcohol consumption on a scale of “never or rarely”; “more than once a month but less than once a week”; “once or twice a week”; “most days”; or “every day.” Due to the small number of participants reporting daily consumption, “most days” and “every day” were collapsed. Participants were then asked how many standard drinks of alcohol they usually consumed on these occasions, and were given examples of what a standard drink of wine, beer, or spirits would be. Average amount consumed in one week was estimated from these responses, and the final dose variable was calculated based upon CDC guidelines (National Center for Health Statistics, 2018): Non-drinkers drank less than 12 drinks per year; light to moderate drinkers consumed at least one drink per month up to seven drinks per week for women and 14 for men; heavy drinkers consumed more than seven drinks per week for women and more than 14 for men. Alcohol data provided by the NHS calculated weekly consumption based upon the number of drinks consumed on the 3 days prior to the interview, together with the number of days alcohol was consumed that week. Number of days consuming 11 or more drinks in one sitting was used as a measure of binge drinking.

### 2.6 Demographic variables

A demographic survey incorporated variables such as highest level of education attained, income, main language spoken in the home, age, and sex. Data extracted from the NHS included age, sex, primary language spoken in the home, gross weekly personal income in deciles, frequency of alcohol consumption in the last 12 months, and estimated total weekly consumption of alcohol.

### 2.7 Statistical analysis

Survey data were analyzed using IBM SPSS version 29 and STATA Version 18. Normality was evaluated using the Shapiro-Wilk test. Descriptive data were analyzed using Means and standard deviations. For inferential analyses, significance was set at *p* ≤ 0.05. Chi square analyses determined relationships between categorical variables. General Linear Models (GLM) were conducted due to the high number of categorical predictor variables and the lack of normality observed in continuous variables. Each age-adjusted neurocognitive domain was included as an independent variable in separate GLM models. Smoking was not included as a potential covariate, as only two participants were current smokers and over 80% had never smoked. Model 1 was adjusted for gender only. Model 2 was adjusted for gender and education level. Model 3 was adjusted for gender, income, and primary language spoken in the home.

The National Health Survey data provides one person weight and 60 replicate weights. Jackknife weighting was conducted using these weighting variables in order to control for individual and sampling characteristics. Descriptive data and chi square analyses were then conducted and displayed using estimated population proportions.

## 3 Results

**FIGURE 1:**
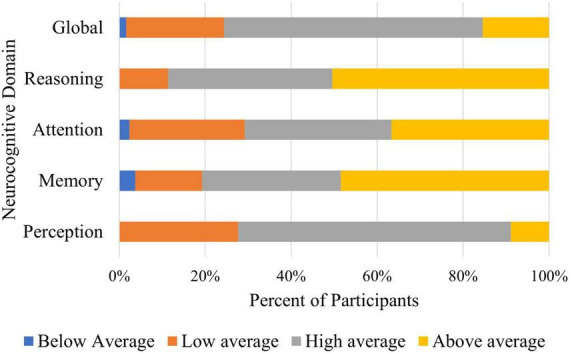
Neurocognitive domain scores. Percentage of participants within each category of cognitive functioning.

Score ranges for the neurocognitive domains are displayed in Figure 1. Mean scores are included in Table 1. Due to reported technical issues with manipulation of the mouse and touchscreen, and substantial variance in the final score, the neurocognitive domain of coordination was deemed unreliable and removed from the analysis.

### 3.1 General linear models (GLM)

**FIGURE 2:**
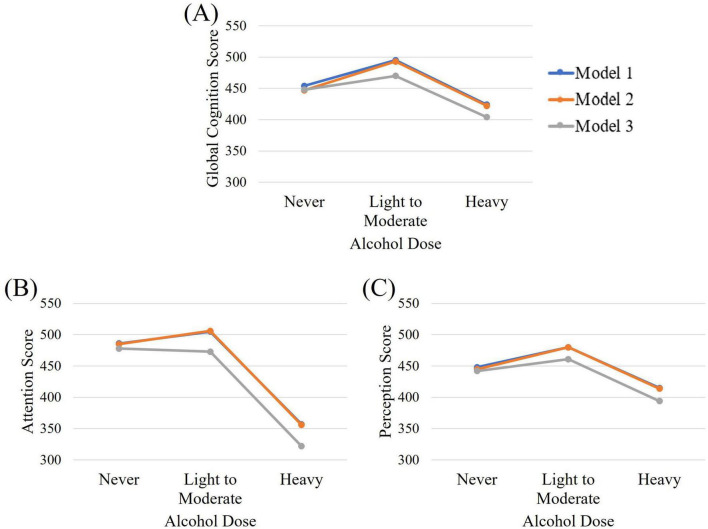
Estimated marginal means for **(A)** global cognition, **(B)** attention, and **(C)** perception across alcohol dose groups when controlling for gender (Model 1), gender and education (Model 2), and gender, income, and language (Model 3).

**FIGURE 3:**
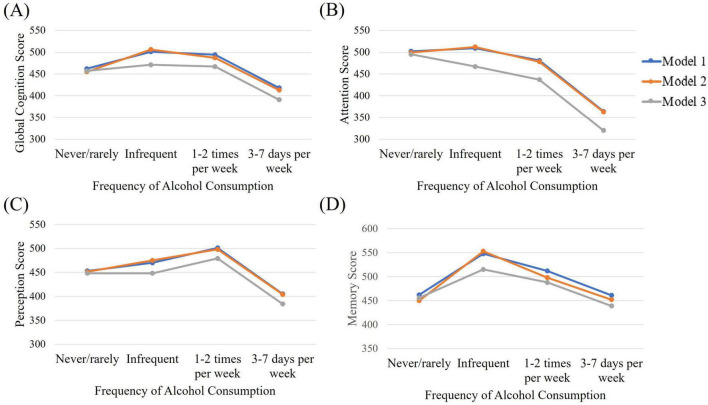
Estimated marginal means for **(A)** global cognition, **(B)** attention, **(C)** perception, and **(D)** memory across alcohol frequency of consumption groups when controlling for gender (Model 1), gender and education (Model 2), and gender, income, and language (Model 3).

The impact of alcohol frequency and dose were investigated separately for their impact on neurocognitive domains. Model 1 controlled for gender, although results were similar when gender was not controlled for. Neither dose nor frequency of alcohol consumption were significantly related to Reasoning. Table 2 highlights the differences between different levels of dose and frequency on all other outcome variables when adjusted for gender; gender and education, and; gender, income and language. Figures 2, 3 are a visual representation of the Estimated Marginal Means. These graphs highlight the reduction in the difference between light and moderate drinkers and infrequent or non-drinkers, and show an increased impact of heavy consumption on cognition, when controlling for income and language. For the results of variables adjusted for income and language separately (see Supplementary Table 1). Reference categories were chosen to optimally highlight the relationships between variables.

**TABLE 2:** General linear model (GLM) results for the relationship between alcohol consumption and neurocognitive domain scores adjusted for demographic covariates.

Items	Model 1				Model 2				Model 3			
	**β**	**95% CI**	***P*-value**	**ηp^2^**	**β**	**95% CI**	***P*-value**	**ηp^2^**	**β**	**95% CI**	***P*-value**	**ηp^2^**
**Global cognition**												
**Alcohol dose**	–	v	0.04	0.07	–	–	0.03	0.06	–	–	0.11	0.04
Abstinent	−40.51	−83, 1.98	0.06	0.03	−45.34	−87.56, −3.11	0.04	0.04	−22.82	−66.72, 21.08	0.31	0.01
Light to moderate	Ref	Ref	Ref	Ref	Ref	Ref	Ref	Ref	Ref	Ref	Ref	Ref
Heavy	−70.3	−135.66, −4.95	0.04	0.04	−70.65	−135.3, −6.01	0.032	0.04	−66.58	−131.61, −1.56	0.045	0.03
**Alcohol frequency**	–	–	0.08	0.06	–	–	0.043	0.07	–	–	0.15	0.05
Never or rarely	−39.82	−89.74, 10.1	0.12	0.02	−50.66	−100.77, −0.55	0.048	0.03	−13.94	−65.77, 37.89	0.6	0
Infrequently	Ref	Ref	Ref	Ref	Ref	Ref	Ref	Ref	Ref	Ref	Ref	Ref
1–2 days per week	−7.19	−64.47, 50.08	0.8	0	−19.55	−77, 37.89	0.5	0	−3.87	−61.27, 53.53	0.89	0
3–7 days per week	−83.94	−156.11, −11.78	0.02	0.04	−92.86	−164.43, −21.3	0.01	0.05	−79.21	−151.6, −6.82	0.03	0.04
**Attention**												
Alcohol dose	–	–	0.01	0.08	–	–	0.01	0.08	–	–	< 0.01	0.09
Abstinent	129.31	34.14, 224.49	0.01	0.06	128.34	32.31, 224.37	0.01	0.06	155.79	57.86, 253.8	< 0.01	0.08
Light to moderate	148.62	58.27, 238.98	< 0.01	0.08	149.68	58.76, 240.6	0	0.08	150.28	59.78, 240.78	< 0.01	0.09
Heavy	Ref	Ref	Ref	Ref	Ref	Ref	Ref	Ref	Ref	Ref	Ref	Ref
Alcohol frequency	–	–	0.03	0.08	–	–	0.02	0.08	–	–	< 0.01	0.11
Never or rarely	137.77	44.84, 230.69	< 0.01	0.07	136.52	43.11, 229.93	0.01	0.07	174.31	80.18, 268.44	< 0.01	0.11
Infrequently	144.56	44.25, 244.87	0.01	0.07	149.81	48.45, 251.18	0	0.07	146.22	45.96, 246.49	0.01	0.07
1–2 days per week	117.21	16.45, 217.96	0.02	0.04	115.35	14.03, 216.66	0.03	0.04	116.91	18.77, 215.06	0.02	0.05
3–7 days per week	Ref	Ref	Ref	Ref	Ref	Ref	Ref	Ref	Ref	Ref	Ref	Ref
**Perception**												
Alcohol dose	–	–	0.05	0.05	–	–	0.04	0.05	–	–	0.08	0.04
Abstinent	33.21	−29.36, 95.79	0.3	0.01	31.28	−31.16, 93.72	0.32	0.01	48.44	−16.43, 113.3	0.14	0.02
Light to moderate	65.28	5.87, 124.68	0.03	0.04	66.4	7.28, 125.52	0.03	0.04	66.72	6.77, 126.66	0.03	0.04
Heavy	Ref	Ref	Ref	Ref	Ref	Ref	Ref	Ref	Ref	Ref	Ref	Ref
Alcohol frequency	–	–	0.03	0.07	–	–	0.03	0.08	–	–	0.04	0.07
Never or rarely	−47.46	−92.82, −2.1	0.04	0.04	−46.9	−92.11, −1.71	0.04	0.04	−30.76	−78.58, 17.05	0.21	0.01
Infrequently	−30.89	−82.38, 20.6	0.24	0.01	−22.92	−75.07, 29.24	0.39	0.01	−30.9	−83.29, 21.49	0.25	0.01
1–2 days per week	Ref	Ref	Ref	Ref	Ref	Ref	Ref	Ref	Ref	Ref	Ref	Ref
3–7 days per week	−95.11	−160.27, −29.95	0.01	0.07	−93.09	−158.03, −28.15	0.01	0.07	−95.06	−159.73, −30.38	< 0.01	0.07
**Memory**												
Alcohol frequency	–	–	0.08	0.06	–	–	0.03	0.08	–	–	0.33	0.03
Never or rarely	−86.19	−155.86, −16.52	0.02	0.05	−102.76	−172.12, −33.4	0	0.07	−58.79	−132.29, 14.71	0.12	0.02
Infrequently	Ref	Ref	Ref	Ref	Ref	Ref	Ref	Ref	Ref	Ref	Ref	Ref
1–2 days per week	−35.82	−115.75, 44.12	0.38	0.01	−55.33	−134.84, 24.19	0.17	0.02	−26.65	−108.04, 54.74	0.52	0
3–7 days per week	−87.33	−188.04, 13.38	0.09	0.02	−101.16	−200.22, −2.11	0.05	0.03	−75.57	−178.22, 27.08	0.15	0.02

Model 1, adjusted for gender; Model 2, adjusted for gender and education; Model 3, adjusted for gender, income, and language spoken at home.

### 3.2 Global cognition

A significant benefit of moderate drinking over heavy drinking is observed in Model 1. When this relationship is adjusted for education level, the superiority of light to moderate consumption over heavy consumption is increased and a benefit over no drinking appears. The benefit of moderate consumption over no drinking disappears when we adjust for income and language. Alcohol frequency of 3–7 days per week was significantly worse than drinking 1–2 times per week (β = −76.75, *p* = 0.04, 95% CI = −149.23, −4.27) or infrequent drinking (see Table 2) in Model 1. When education was included in the model, infrequent drinking was also superior to drinking never or rarely. When adjusting for income and language spoken in the home, infrequent and moderate drinking were only superior to heavy drinking, although the model itself was no longer significant.

### 3.3 Attention

Heavy alcohol consumption showed significantly lower scores for attention than did abstinent or moderate consumption, no matter which model was used. The same pattern was seen for frequency of alcohol consumption.

### 3.4 Perception

The relationship between alcohol dose and perception is not significant in Models 1 or 3. Only when adjusting for education and gender does a benefit of moderate consumption become evident. When investigating frequency of consumption, heavy or rare drinking shows worse performance than drinking 1–2 times per week. This pattern is maintained when correcting for education. Model 2 also shows a benefit of infrequent consumption over drinking 3–7 days per week (β = 70.17, *p* = 0.04, 95% CI = 5.2, 135.14). When adjusting for income and language, the benefit of weekly consumption over infrequent or rare consumption was no longer present.

### 3.5 Memory

No relationship was seen between alcohol dose and memory. The relationship between alcohol frequency and memory are not significant in models 1 and 3. When adjusting for gender and education, infrequent consumption had better scores than never or rarely drinking, or drinking 3–7 days per week.

### 3.6 Patterns of alcohol consumption

**FIGURE 4:**
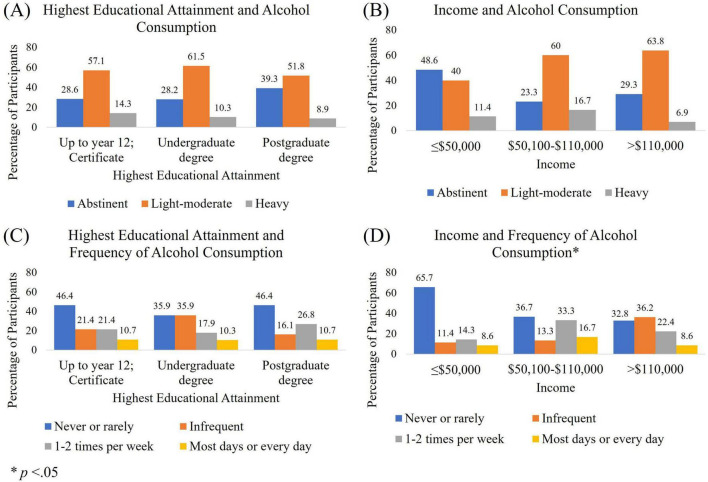
Patterns of alcohol consumption per week. Alcohol per week by **(A)** educational attainment and **(B)** income. Frequency of alcohol consumption dose per week by **(C)** educational attainment and **(D)** income.

**FIGURE 5:**
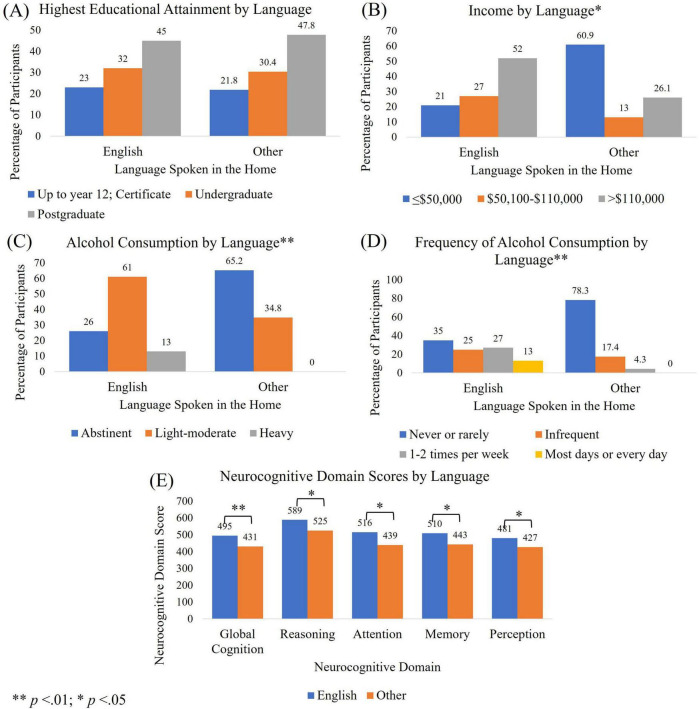
Participant characteristics by language. Differences between individuals speaking English or a language other than English in the home for **(A)** educational attainment; **(B)** income; **(C)** alcohol consumption dose per week; **(D)** frequency of alcohol consumption, and; **(E)** neurocognitive domain scores.

Figure 4 highlights the different patterns of alcohol consumption associated with educational attainment and income. This relationship was only significant for income and alcohol consumption frequency. However, for Chi square analyses to be conducted, income had to be collapsed to above and below $110,000 due to cell sizes smaller than five. Participants who spoke a language other than English in the home had similar educational attainment to those who spoke English. However, their income was significantly lower, and their alcohol consumption was significantly less in terms of dose and frequency (see Figure 5). Participants who spoke a language other than English at home also had significantly lower scores on all neurocognitive domains, despite similar educational attainment. For Chi square analyses described in Figure 5, alcohol dose and consumption were dichotomized into abstinent and other, and never or rarely drink and all other frequencies of consumption due to small cell sizes.

### 3.7 Australian national health survey—2018

Data from the National Health Survey 2018 was used to further investigate the relationships between drinking patterns and socioeconomic and demographic variables. When investigating alcohol consumption across income and education groups, we observed an even more pronounced difference in patterns of drinking (see Supplementary Figure 1). Higher income respondents were more likely to drink heavily and frequently than low income, but those with higher educational qualifications had similar drinking patterns to other non-school qualifications. Those with certificate training had the highest rates of daily or almost daily drinking. We also investigated the relationship between income and binge drinking, which has often been associated with poorer cognitive and physical health outcomes. A U-shaped curve was observed with middle-quintile income earners binge drinking more frequently than those at the lower or higher ends of the income spectrum. Binge drinking is also more associated with lower educational attainment. Language spoken in the home had a similar pattern to the data found in the cross-sectional survey data (see Supplementary Figure 2). Those who spoke a language other than English in the home were more likely to have attained a bachelor or postgraduate qualification, were more likely to be in the lowest two income quintiles, and consumed alcohol less frequently and in smaller doses.

## 4 Discussion

The present study aimed to evaluate the relationship between alcohol consumption and cognition, investigating the differential influence of potential confounders: education; income; and language. Heavy and frequent alcohol consumption was associated with poorer performance on attention, perception, and global cognition. When including education as a separate covariate, results were more likely to indicate a benefit of light to moderate alcohol consumption, or of moderately frequent consumption over abstinence or heavy drinking. A difference between drinking frequencies on memory scores was only significant when education was controlled for. Benefits of moderate consumption were not observed when adjusted for income and language. An assessment of drinking patterns associated with education and income clarify the potential reasons for these differences. Findings from both the cognition study and NHS data show that higher income is associated with higher rates of alcohol consumption. However, individuals with higher educational qualifications have similar drinking and abstinence rates to those with the lowest levels of educational attainment. This could be due to the differences in job opportunities and average income observed in different disciplines of postgraduate study. Findings could also be influenced by the number of highly paid positions in trades that do not require university qualifications.

The findings from the current study indicate little difference between abstinence and moderate drinking in terms of cognitive outcomes. However, several human neuroimaging studies have identified a linear relationship, with greater alcohol consumption associated with smaller volume across a range of brain regions (Karoly et al., 2024). This reduction in cortical thickness was observed even with low and moderate alcohol consumption. Findings from animal models have identified both protective and detrimental impacts of low-moderate consumption. Studies of neuronal cultures found that low ethanol concentrations prevented neurodegeneration due to β-amyloid and other neuroinflammatory proteins (Collins et al., 2010; Muñoz et al., 2015). This could potentially explain the reduction in dementia risk observed in some human studies. On the other hand, a study of Sprague-Dawley rats consuming moderate amounts of alcohol found a reduction in the number of cells produced in the dentate gyrus of the hippocampus, despite no significant reductions in behavioral tests of motor skills or learning (Anderson et al., 2012). Similarly, human cognitive testing may fail to identify early or minimal neurological impairment caused by alcohol consumption.

Individuals from lower SES experience disproportionate levels of alcohol-related harm (Katikireddi et al., 2017), greater mortality risk (Stringhini et al., 2017), and accelerated rates of cognitive decline (Steptoe and Zaninotto, 2020) compared to their more affluent counterparts. The fact that lower SES groups also have the highest rates of abstinence could bias study findings in favor of any amount of alcohol consumption over abstinence. Data from the NHS supports previous research suggesting that impairments observed in those from lower SES who consume less frequent, and less weekly doses of alcohol, may be due to infrequent episodes of “binge” drinking (Probst et al., 2020). Previous studies have also found long-term binge drinking associated with impaired memory, learning, and planning (Hendriks et al., 2020). It is unclear how infrequent, heavy episodes of drinking are captured in studies that reflect only daily or weekly drinking habits.

Language was also strongly associated with drinking behaviors, SES, and cognitive test performance. Despite holding higher educational qualifications, individuals who spoke a language other than English at home were more likely to score worse on all cognitive domains, creating additional bias in favor of alcohol consumption if not controlled for. Past research has also identified potential enhancements in cognitive performance (specifically processing speed) in those who are bi- or multi-lingual (Pacifico et al., 2023), suggesting that English proficiency may be a better predictor of cognitive test performance than primary language spoken. However, the relationship between language proficiency, alcohol consumption and SES must be investigated further. Conducting neurocognitive testing in participants’ first language may help to overcome this confounding and produce more reliable results.

Previous studies have identified sex-specific deficits in cognitive functions associated with alcohol consumption (Hone et al., 2020; Salvador et al., 2022). However, the current study found that gender had little influence on the relationship between alcohol consumption and cognitive outcomes. This may be due to the small number of male participants in the sample. It could also be due to other socio-economic or lifestyle factors that could not be adequately explored due to the limited sample size.

## 5 Strengths and limitations

The study utilized an adequately powered cross-sectional survey and a large population-based dataset to test the hypotheses. Findings from the survey were supported by the relationships identified in NHS data, indicating the generalizability of the results. Limitations of the study included the small number of questions regarding alcohol consumption and the lack of questions investigating past drinking behaviors. However, an investigation of past drinking behaviors was frequently omitted from studies that identified links between moderate alcohol consumption and enhanced cognitive function, making our paper comparable in methodology to the papers our findings challenged; enabling us to replicate and then test their findings. The sample contained a large number of highly educated participants, and few heavy or frequent drinkers. The findings also rely on participant recall. Recall bias is commonly seen in alcohol questionnaires, particularly in sporadic drinkers, with underestimation of consumption also common (Gmel and Daeppen, 2007; Gilligan et al., 2019). While the study was adequately powered for the included analyses, a larger sample may have allowed for a larger proportion of male participants and a more diverse sample in terms of socio-economic and cultural backgrounds that would have enabled additional investigation relating to the influences of these factors.

## 6 Conclusion

Our findings support previous assertions that income may provide a superior measure of SES than education. This is evidenced by the fact that education and income are associated with substantially different patterns of alcohol consumption. The relationships between income and education, and other lifestyle factors (such as exercise, nutrition, smoking, or occupation), should also be investigated to further explore the suitability of these variables as indicators of SES in health research. The study also identified detrimental impacts of heavy and/or frequent drinking on cognitive function, with little evidence for any benefit of moderate consumption over abstinence when income and language are controlled for. The study highlights a number of common study characteristics that may bias outcomes toward showing a beneficial impact of moderate drinking behaviors on cognitive function. Future research investigating lifestyle factors and cognitive function should include additional measures of SES to ensure they are appropriately controlling for confounding factors. Research that fails to account for the influence of accurate SES measures may unintentionally lead to an increase in damaging drinking behaviors and alcohol-related injury and disease. Understanding the true impact of alcohol on physical and cognitive health is crucial for developing guidelines and public health policy for safe alcohol consumption.

## Data Availability

The raw data supporting the conclusions of this article will be made available by the authors upon reasonable request.
