# Supplementary material for: Moderate alcohol consumption does not protect cognitive function when controlling for income and cultural factors
Source: Front Aging Neurosci. 2025 May 26;17:1569069. doi: 10.3389/fnagi.2025.1569069 (PMC12146365; doi:10.3389/fnagi.2025.1569069)
Supplement: Supplementary file 1 [file Table_1.docx]

Supplementary Material

# Supplementary Table 1

**Supplementary Table 1.** General Linear Model (GLM) results for the relationship between alcohol consumption and neurocognitive domain scores adjusted for income and primary language spoken in the home.

|  | | **Model 4 - Adjusted for gender and income** | | | | **Model 5 - Adjusted for gender and primary language spoken in the home** | | | |
| --- | --- | --- | --- | --- | --- | --- | --- | --- | --- |
|  |  | ***β*** | **95% CI** | **P value** | **Ƞp^2^** | ***β*** | **95% CI** | **P value** | **Ƞp^2^** |
| **Global Cognition** | | | | | | | | | |
| **Alcohol Dose** | |  |  | 0.11 | 0.04 |  |  | 0.05 | 0.05 |
|  | Abstinent | -32.18 | -75.09, 19,74 | 0.14 | 0.02 | -25.79 | -69.56, 17.98 | 0.25 | 0.01 |
|  | Light to moderate | REF | REF | REF | REF | REF | REF | REF | REF |
|  | Heavy | -59.97 | -125.08, 5.15 | 0.07 | 0.03 | -76.46 | -140.97, -11.95 | 0.02 | 0.05 |
| **Alcohol frequency** | |  |  | 0.19 | 0.04 |  |  | 0.08 | 0.06 |
|  | Never or rarely | -22.94 | -74.11, 28.23 | 0.38 | 0.01 | -26.75 | -76.95, 23.45 | 0.29 | 0.01 |
|  | Infrequently | -1.97 | -59.66, 55.72 | 0.95 | <.01 | REF | REF | REF | REF |
|  | 1-2 days per week | REF | REF | REF | REF | -13.21 | -69.65, 43.22 | 0.64 | <.01 |
|  | 3-7 days per week | -72.89 | -144.48, -1.31 | 0.05 | 0.03 | -92.08 | -163.22, -20.94 | 0.01 | 0.05 |
| **Attention** | | | | | | | | | |
| **Alcohol Dose** | |  |  | 0.011 | 0.074 |  |  | <.01 | 0.1 |
|  | Abstinent | 129.75 | 33.99, 225.51 | 0.01 | 0.06 | 160.25 | 63.53, 256.87 | <.01 | 0.08 |
|  | Light to moderate | 139.5 | 48.45, 230.54 | <.01 | 0.07 | 157.74 | 68.84, 246.65 | <.01 | 0.1 |
|  | Heavy | REF | REF | REF | REF | REF | REF | REF | REF |
| **Alcohol frequency** | |  |  | 0.02 | 0.08 |  |  | <.01 | 0.11 |
|  | Never or rarely | 148.18 | 55, 241.36 | <.01 | 0.08 | 172.28 | 78.63, 265.93 | <.01 | 0.1 |
|  | Infrequently | 132.08 | 30.68, 233.49 | 0.01 | 0.05 | 157.8 | 59.79, 255.82 | <.01 | 0.08 |
|  | 1-2 days per week | 112.73 | 12.81, 212.65 | 0.03 | 0.04 | 120.65 | 22.63, 218.67 | 0.02 | 0.05 |
|  | 3-7 days per week | REF | REF | REF | REF | REF | REF | REF | REF |
| **Perception** | | | | | | | | | |
| **Alcohol Dose** | |  |  | 0.1 | 0.04 |  |  | 0.06 | 0.05 |
|  | Abstinent | 32.93 | -30.3, 96.15 | 0.3 | 0.01 | 51.08 | -12.87, 115.02 | 0.12 | 0.02 |
|  | Light to moderate | 60.29 | 0.18, 120.4 | 0.05 | 0.03 | 70.54 | 11.76, 129.32 | 0.02 | 0.05 |
|  | Heavy | REF | REF | REF | REF | REF | REF | REF | REF |
| **Alcohol frequency** | |  |  | 0.05 | 0.07 |  |  | 0.03 | 0.07 |
|  | Never or rarely | 51.07 | -9.76, 111.9 | 0.1 | 0.02 | 64.51 | 3.09, 125.93 | 0.04 | 0.04 |
|  | Infrequently | 57 | -9.2, 123.2 | 0.09 | 0.02 | 70.69 | 6.41, 134.97 | 0.03 | 0.04 |
|  | 1-2 days per week | 92.94 | 27.71, 158.18 | <.01 | 0.06 | 96.79 | 32.5, 161.08 | <.01 | 0.07 |
|  | 3-7 days per week | REF | REF | REF | REF | REF | REF | REF | REF |
| **Memory** | | | | | | | | | |
| **Alcohol frequency** | |  |  | 0.28 | 0.03 |  |  | 0.14 | 0.05 |
|  | Never or rarely | -64.66 | -137.47, 8.16 | 0.08 | 0.03 | -74.89 | -145.97, -3.81 | 0.04 | 0.04 |
|  | Infrequently | REF | REF | REF | REF | REF | REF | REF | REF |
|  | 1-2 days per week | -21.78 | -102.78, 59.22 | 0.6 | <.01 | -41.02 | -120.92, 38.88 | 0.31 | 0.01 |
|  | 3-7 days per week | -68.65 | -170.65, 33.35 | 0.19 | 0.02 | -94.36 | -195.09, 6.37 | 0.07 | 0.03 |

CI, Confidence Interval; REF, Reference group

# Supplementary Figure 1


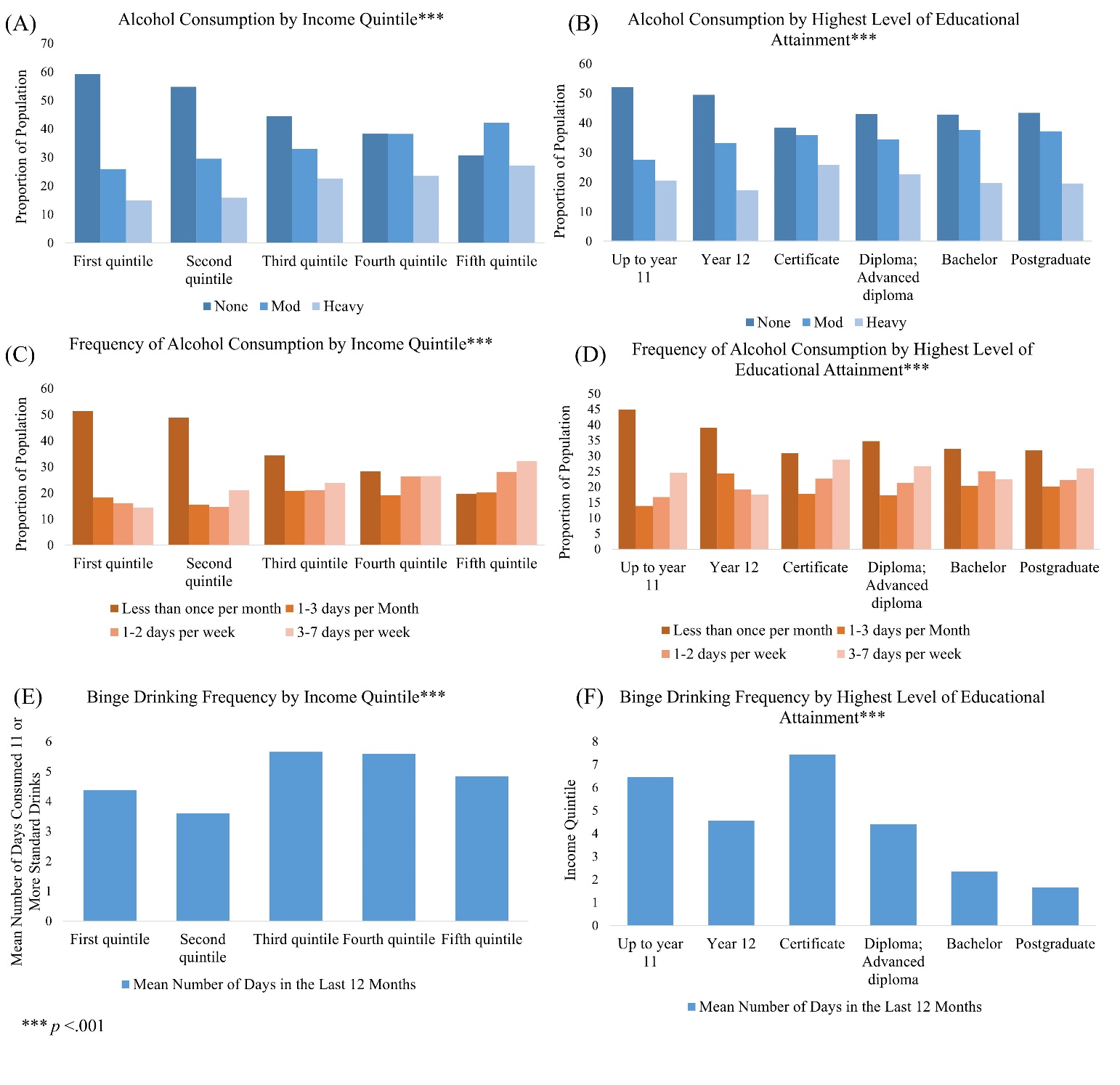


# Supplementary Figure 1. Patterns of alcohol consumption by (A) income and (B) educational attainment. Frequency of alcohol consumption by (C) income and (D) educational attainment. Mean number of days individuals consumed 11 or more drinks in one sitting by (E) income and (F) educational attainment.

## Supplementary Figure 2


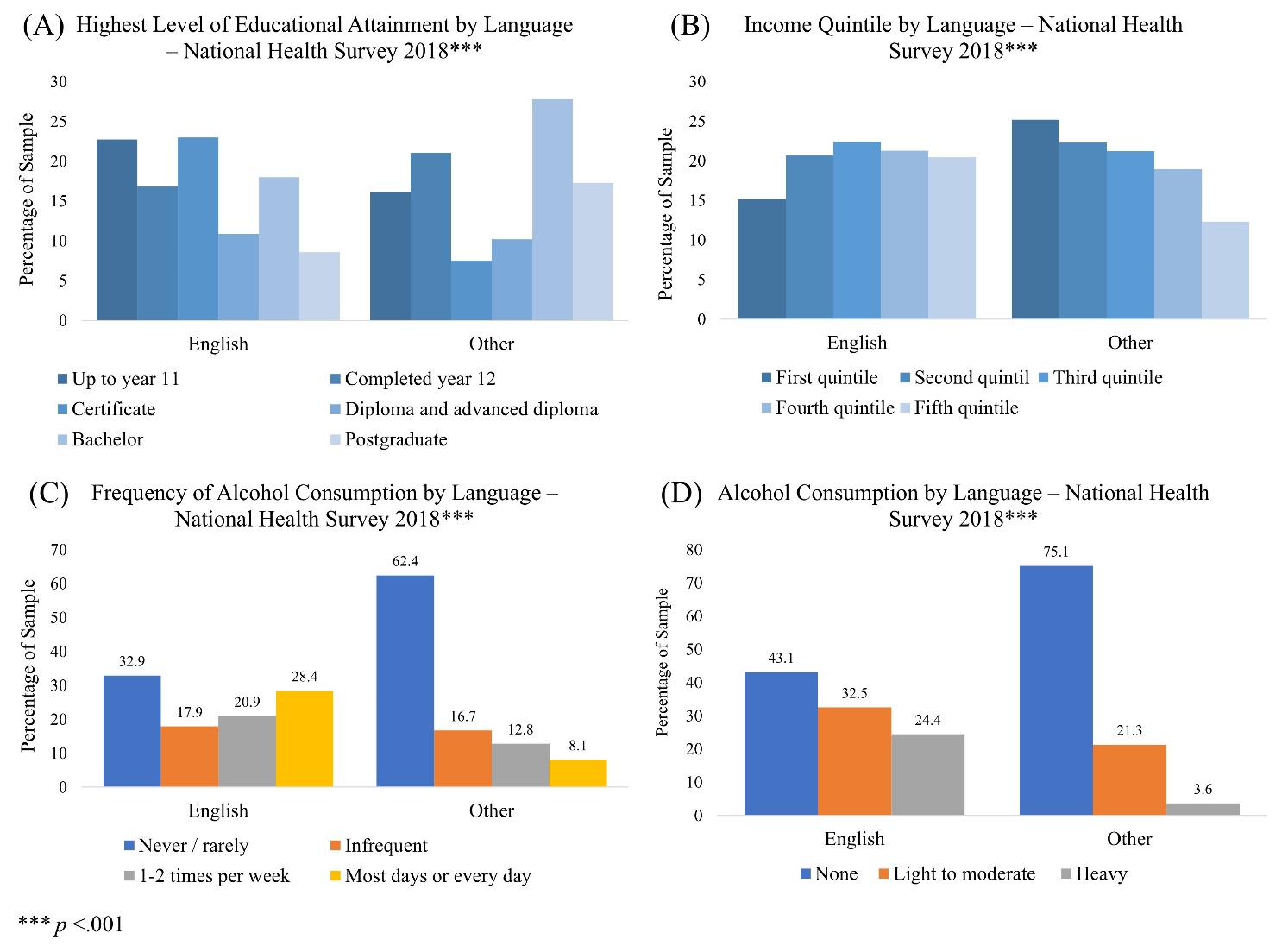


**Supplementary Figure 2.** Differences between individuals speaking English or a language other than English in the home for (A) educational attainment, (B) income, (c) frequency of alcohol consumption, and (D) alcohol consumption dose per week.
